# Alcoholic Hyperæsthesia and Paræsthesia of the Feet

**Published:** 1885-05-20

**Authors:** C. F. Barker

**Affiliations:** Fort Hamilton, N. Y.


### ALCOHOLIC HYPERÆSTHESIA AND PARÆSTHESIA OF THE FEET.

BY C. F. BARKER, M.D., Fort Hamilton, N. Y.

As the various effects of alcohol, its treatment, causes, etc., are at present causing more or less discussion among the profession, I take the liberty of narrating the various features of these cases as they have presented themselves to me. It has been my good fortune to have been able to observe and treat many ot them.

This affection does not, as a rule, occur to those who are in the habit of drinking periodically, but it is the steady imbiber, he who has habituated himself or herself to the use of stimulants for months and years. It has no choice as to age, attacking the young as well as the old. We find it appearing in the phlegmatic as often as in the nervous patient. Like many of the affections appearing in the inebriate, it usually is first noticed when the victim begins to lessen his or her amount of stimulants. This, however, does not always hold true, as you will find those in whom the disease has been complained of, while the sufferer was markedly under the influence of liquor, and again sometimes attacks the patients while they are far advanced in convalescence. I have in mind a case where the symptoms did not show themselves for weeks after the gentleman had been without stimulants, and another where the invasion was apparent while the man was still continuing his debauch. In the latter case, the attending physician mistook the symptoms for those of rheumatism. Quality or class of liquors does not seem to have any special bearing as to the trouble. The premonitory symptoms are not the same in any two cases. In some the disease is first noticed by the patient on rising in the morning ; the feeling as if they had had a bruise from stepping on a stone, or, perhaps, muscular twitchings may be the first inconvenience experienced by the patient. These disagreeable feelings wear away during the day, but gradually become more marked and troublesome. After the disease is fairly developed they are often obliged to remain in bed. They suffer from pains of varying degress of severity. The intense pains last but a few days, while the milder forms continue for weeks. The patient is often unable to stand the weight of the bedclothes upon the troublesome parts, and to have the shoes and stockings on is impossible. At times the slightest touch is so painful that the physician is unable to examine the parts. The toes are of much annoyance to the patients, having, as they will say, the feeling of being drawn under the feet This sensation is so marked that patients will ofttimes feel with their hands to satisfy themselves that they are really in proper position. The soles of the feet usually give much trouble and are last to yield to treatment. The feeling is as if pins and needles were sticking into them, or that heavy weights were fastened to their shoes. In the more severe cases, if you have an opportunity to watch these patients when they alight from their bed, you will observe that they move as one does when he is carefully feeling his way in the dark. They seem to be afraid to step out. When asked why they do not move their feet more naturally, they will tell you they are not sure that they will be able to stand on them if raised and put down in the ordinary manner.

At night more often than during the day they experience muscular twitchings and a sensation of burning (not like that produced by an electric current), but as if they were stepping on a hot stove, a disagreeable sense of heat. These various feelings cause them to lose sleep and quite often interfere with their appetite. For the same reason that they do not use their feet in walking, they are not able to go up and down stairs, without aid. To go up stairs is more difficult than to descend. In a case lately under my care the patient was troubled with pains, taking a serpentine course from the ankle to all parts of the foot. They would not follow the course of any nerve, but dart in this irregular manner from one point to another, causing the patient much distress. It is exceedinly rare to have these sensations extend above the middle of the belly of the gastrocnemius muscle.

I have heard of cases where this disease has been mistaken for rheumatism and the patient placed under treatment for that malady.

Under proper care and treatment these patients recover perfectly. The recovery is slow, depending greatly upon the condition of the patient. Relapses do not occur unless the patient returns to his old ways of dissipation, and then but little over-indulgence will cause a speedy return.

Various modes and measures have been tried and advanced for the treatment of this affection, and to enumerate them all in this article would occupy too much valuable room. Those which have proved most efficacious are electricity, massage, hot and cold water baths, moderate exercise, to those who are able to be about, and tonics.

There is no doubt but that electricity takes the first position in the treatment of this disease. Both galvanism and faradism have yielded excellent results, but for my own use, taking the experience which has been afforded me as a guide, I much prefer the faradic current, in strength varying according to the severity of the case. The little French instrument, "Gaiffe battery," has in some cases proved very useful, but as it is limited in its power it cannot be used in all cases. The amount of time employed at each sitting varies ; usually, to commence with, from ten to fifteen minutes is as much as the patient will be able to bear, but as improvement takes place the sittings may be increased. After the patients have become accustomed to the sensations of the battery, they are anxious to have it applied, as relief is experienced which is very marked. If applied before bedtime it gives the subject a much better chance of having a good night's rest than when used in the morning.

Massage, to those who can bear the process, is found often of great service in giving relief.

Bathing the parts in hot or cold water, hot water usually being preferred, night and morning, has been found to relieve many where massage could not be practiced. The temperature of the water should be that which is most agreeable to the patient.

As to the choice of drugs, that depends greatly upon the condition of the patient. The employment of the sulphate of quinine has met with most pleasing results in my hands, and its value, I think, will be found by those who will try it. Pil. phos. ferri, quinia et strychnia has also been of much service. Nux vomica in the form of the tincture has also proved useful.

Much more could be written regarding this disease, but the foregoing may be a nucleus upon which to build. Advancing step by step as we are in the subject of inebriety and its complications, we may soon be able to diagnosticate and treat with equal certainty its many forms as we do the now classified diseases.—*New York Medical Record.*

